# Assessing the influence of microwave-assisted synthesis parameters and stabilizing ligands on the optical properties of AIS/ZnS quantum dots

**DOI:** 10.1038/s41598-022-25498-3

**Published:** 2022-12-20

**Authors:** Lorena Dhamo, K. David Wegner, Christian Würth, Ines Häusler, Vasile-Dan Hodoroaba, Ute Resch-Genger

**Affiliations:** 1 Division Biophotonics, Federal Institute for Materials Research and Testing (BAM), 12489 Berlin, Germany; 2 Departments of Physics, Humboldt Universität Zu Berlin, 12489 Berlin, Germany; 3 Division Surface Analysis and Interfacial Chemistry, Federal Institute for Materials Research and Testing (BAM), 12203 Berlin, Germany

**Keywords:** Materials science, Nanoscience and technology

## Abstract

Luminescent semiconductor quantum dots (QDs) are frequently used in the life and material sciences as reporter for bioimaging studies and as active components in devices such as displays, light-emitting diodes, solar cells, and sensors. Increasing concerns regarding the use of toxic elements like cadmium and lead, and hazardous organic solvents during QD synthesis have meanwhile triggered the search for heavy-metal free QDs using green chemistry syntheses methods. Interesting candidates are ternary AgInS_2_ (AIS) QDs that exhibit broad photoluminescence (PL) bands, large effective Stokes shifts, high PL quantum yields (PL QYs), and long PL lifetimes, which are particularly beneficial for applications such as bioimaging, white light-emitting diodes, and solar concentrators. In addition, these nanomaterials can be prepared in high quality with a microwave-assisted (MW) synthesis in aqueous solution. The homogeneous heat diffusion and instant temperature rise of the MW synthesis enables a better control of QD nucleation and growth and thus increases the batch-to-batch reproducibility. In this study, we systematically explored the MW synthesis of AIS/ZnS QDs by varying parameters such as the order of reagent addition, precursor concentration, and type of stabilizing thiol ligand, and assessed their influence on the optical properties of the resulting AIS/ZnS QDs. Under optimized synthesis conditions, water-soluble AIS/ZnS QDs with a PL QY of 65% and excellent colloidal and long-term stability could be reproducible prepared.

In the last decades, semiconductor nanocrystals (also termed quantum dots, QDs) became popular for applications such as bioimaging, biosensing, and optoelectronic devices^1–6^. The strong interest in QDs is based on the possibility to control their optical properties by their size, shape, and chemical composition. Moreover, QDs possess very high photoluminescence (PL) quantum yields (QYs) and a high photostability^7–9^. The most popular QDs used to be based either on the heavy-metal cadmium or lead^10,11^. Meanwhile, the potential toxicity of these heavy metals has raised considerable concerns about their use in commercial devices and applications particularly in Europe. Moreover, except for CdTe, high quality II/VI and IV/VI QDs as well as less toxic III/V QDs such as InP are commonly synthesized in environmentally hazardous organic solvents^12^. Therefore, with the increasing pressure to develop and apply greener chemistry principles and safe-by-design approaches for nanomaterials, in the last years, researchers started to focus on alternatives for these QDs, which still possess comparably beneficial optical properties such as high PL QY values but are heavy-metal free. This has triggered the interest in InP^13^, carbon dots^14,15^, silicon QDs^16^ as well as ternary QDs like CuInS_2_ (CIS) and AgInS_2_ (AIS) or quaternary QDs like AgInSZn (AISZ) and ZnCuInS (ZCIS) with PL in the visible and near infrared (NIR) region^17^. In contrast to binary QDs such as II/VI, IV/IV, and III/V QDs, where the optical properties are solely controlled by the width of the band gap, the PL properties of ternary QDs are ascribed to defect states in the band gap structure^18^. This leads to a large effective Stokes shift, broad PL bands, and long PL lifetimes in the order of a few hundred nanoseconds. Different PL mechanisms such as the radiative recombination of donor–acceptor (D–A) pairs, a self-trapped exciton model (STE), recombination of a localized hole with a conduction band electron, and a combination of these mechanisms have been used to explain the PL of ternary QDs^19–21^.

Like their binary QD counterparts, ternary QDs are typically prepared by hot injection or heating-up methods in high boiling organic solvents utilizing ligands such as 1-dodecanthiol or oleylamine. The application of these hydrophobic QDs in biologically relevant environments thus requires a post-modification step to render these hydrophobic QDs water dispersible. A popular approach is the exchange of the native hydrophobic ligands for hydrophilic ligands such as glutathione (GSH), mercaptoacetic acid (MAA) or 3-mercaptopropionic acid (MPA). This ligand exchange can result in a considerable diminution of PL QY caused by the formation of new surface defect states^22^. In addition, the synthesis in organic solvents is not environmentally friendly and does not meet the principles of green chemistry. To lower the impact on the environment, the direct synthesis of ternary QDs in aqueous media using fewer toxic reagents is desired^23,24^. Therefore, next to classical wet-synthesis approaches^25–27^ and hydrothermal methods^28,29^, microwave-assisted (MW) syntheses increased in popularity for the preparation of different types of nanomaterials^30^. Utilizing MW radiation has many advantages like a very fast increase of the reaction temperature and a stable thermal gradient in the reaction mixture. This provides a more uniform nanoparticle preparation and increases the reproducibility of the reaction^31–34^. Although MW synthesis approaches have been meanwhile optimized for binary QDs^35,36^, there are only few reports on the fabrication of ternary QDs with PL QYs > 50%^37–41^. The strong influence of different synthesis parameters for a MW synthesis of AIS QDs was recently shown by Soares et al.^42^, who used a design-of-experiment approach to rationally prepare AIS/ZnS QDs with precisely tuned PL features.

In this work, we aimed for a deeper insight into the MW synthesis of AIS QDs and assessed synthesis parameters such as the ratio of the precursors and sequence of precursor addition as well as the role of commonly employed surface ligands such as GSH, MPA, thioglycolic acid (TGA), and sodium 3-mercapto-1-propanesulfonic acid (MPSA) on AIS QD formation. By evaluating the PL properties of the resulting AIS QDs, we could show that even the sequence of precursor addition can play an important role for the preparation of AIS/ZnS QDs with high PL QY values of ca. 65% using MPA or GSH as stabilizing ligands.

In this work, we aimed for a deeper insight into the MW synthesis of AIS QDs and assessed synthesis parameters such as the ratio of the precursors and sequence of precursor addition as well as the role of commonly employed surface ligands such as GSH, MPA, thioglycolic acid (TGA), and sodium 3-mercapto-1-propanesulfonic acid (MPSA) on AIS QD formation. By evaluating the PL properties of the resulting AIS QDs, we could show that even the sequence of precursor addition can play an important role for the preparation of AIS/ZnS QDs with high PL QY values of ca. 65% using MPA or GSH as stabilizing ligands.

## Materials and methods

### Chemicals

Silver nitrate (Honeywell, 99.8%), indium(III) chloride (Sigma-Aldrich, 98%), L-reduced glutathione (Sigma-Aldrich), 3-mercaptopropionic acid (AppliChem), sodium sulphide (Abcr, 98%), ammonium hydroxide 5 M (Sigma-Aldrich), citric acid (Roth, 99.5%), zinc acetate (Chemsolute, 99.5%), ethanol (Merck, absolute), sodium 3-mercaptopro-1-propanesulfonic acid (ChemPUR), thioglycolic acid (Merck) were used in the present work without additional purification.

## Methods

### TEM measurements

The TEM measurements were recorded at the Technical University of Berlin (TU Berlin) on the FEI Titan 80–300 located there and fast Fourier transform pattern were used to evaluate the diffraction patterns of the obtained QDs. The images were taken at 300 kV.

### EDX measurements

Energy-Dispersive X-ray Spectroscopy (EDX) measurements were performed with a Thermo Fisher Scientific system (Waltham, MA, USA) equipped with an UltraDry silicon-drift detector (SDD) with a nominal detector area of 100 mm^2^. The EDX detector was attached to a Scanning Electron Microscope (SEM) of type Zeiss Supra 40 (Zeiss, Oberkochen, Germany) with a Schottky-field emitter. The software Pathfinder 1.3 was employed for the measurements and data analysis. Each sample was analyzed on three different areas of about 100 × 100 µm^2^ at an excitation energy of 10 keV and a mean value for the elemental composition was calculated for each sample using a standardless quantification. The S Kα (2.31 keV), Zn Lα (1.01 keV), Ag Lα (2.98 keV) and In Lα (3.29 keV) X-ray lines were utilized for quantification.

### Absorption measurements

The absorption spectra of diluted aqueous QD dispersions were measured with a calibrated double-beam spectrometer Specord 210plus (Analytik Jena) in a wavelength range of 200–800 nm using an integration time of 0.5 s/nm.

### Photoluminescence measurements

Steady-state PL emission spectra of diluted aqueous QD dispersions were recorded with a calibrated fluorometer FLS 920 (Edinburgh Instruments) equipped with a Xenon lamp, using an excitation wavelength of 450 nm. The integration time was set to 0.5 s and 3 scans were done per measurement.

### Photoluminescence quantum yields (PL QY)

PL QY values were absolutely determined using the integrating sphere setup Quantaurus-QY C11347-11 (Hamamatsu) previously evaluated by our group^43–45^. The measurements of PL QY of the QD dispersions were performed at room temperature in 10 × 10 mm long neck quartz cuvettes from Hamamatsu.

### PL decay kinetics

The time-resolved PL measurements of the QD dispersions were done with the lifetime spectrofluorometer FLS 920 (Edinburgh Instruments) equipped with a pulsed EPLED laser (wavelength of 375 nm ± 5 nm; pulse duration of 60 ps, pulse repetition rate of 100 kHz, equalling one-pulse every 10 µs), and a MCP detector using time-correlated single-photon counting (TCSPC) detection. The multiexponential PL decay curves, that were always recorded at the emission maximum of the respective QD sample, were evaluated using the software FAST (Edinburgh Instrument). The measured PL decay profiles were used to calculate the luminescence decay times by applying tri-exponential reconvolution fits, thereby considering the instrument response function (IRF) determined with a scatterer at a detection variable from sample to sample (at the max PL emission) from 600 to 720 nm. Amplitude-weighted average decay times were calculated from the fitted multiexponential values using equation S1 given in the Supporting Information (SI).

### AIS/ZnS synthesis

**Figure 1:**
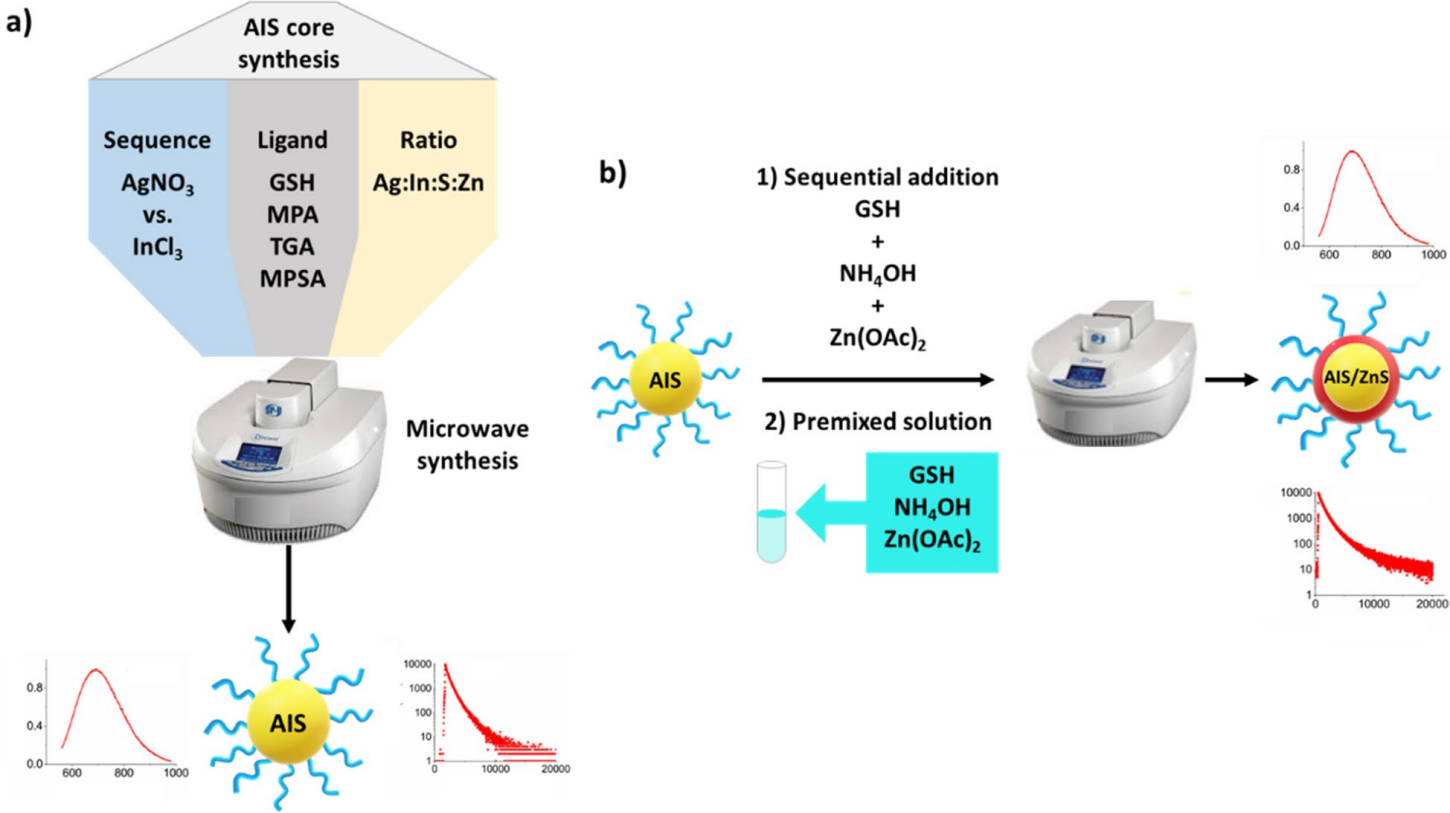
Overview of the different procedures (*C1* and *C2)* as well as varied synthesis parameters for the AIS core preparation (panel **a**) and the schematic presentation of the core/shell synthesis using a sequential addition of reagents (*CS1*) or a premixed solution (*CS2*) (panel **b**).

The MW syntheses of the AIS/ZnS QDs were performed using different protocols that are subsequently referred to as procedures *C1, C2, CS1*, and *CS2* for the core (C) and core/shell (CS) syntheses, respectively. The main difference between the core and core/shell syntheses is highlighted in Fig. 1.

#### Core synthesis C1

500 µL 0.1 M aqueous solution of silver nitrate (AgNO_3_), 1.4 mL 0.5 M aqueous solution GSH, 700 µL 5 M ammonia solution (NH_4_OH), 330 µL 2.0 M aqueous solution citric acid (CA), and 560 µL 1.0 M aqueous solution indium(III) chloride (InCl_3_) (containing 0.1 M nitric acid (HNO_3_)) were added to a MW vessel containing 10 mL deionized water under stirring. The pH of the reaction mixture was adjusted to pH 8–9. Then, 560 µL of a 1.0 M aqueous solution sodium sulphide (Na_2_S) were quickly added and the vessel was heated at 100 °C for 50 min.

#### Core synthesis C2

560 µl 1.0 M aqueous solution InCl_3_ (containing 0.1 M HNO_3_), 1.4 mL 0.5 M GSH, 700 µL 5 M NH_4_OH, and 330 µL 2.0 M aqueous CA were added to a MW vessel containing 10 mL deionized water under stirring, followed by addition of 500 µL 0.1 M aqueous solution AgNO_3_. The pH of the mixture was adjusted to pH 8–9. Then, 560 µL of a 1.0 M aqueous solution Na_2_S were quickly added, and the vessel was heated at 100 °C for 50 min.

#### Shell synthesis CS1

For the ZnS shelling procedure, a solution of 560 µL 0.5 M aqueous GSH, 560 µL 5.0 M NH_4_OH, and 560 µL 1.0 M (containing 0.01 M nitric acid) of an aqueous solution zinc acetate (Zn(OAc)_2_) was added to the dispersion containing the core AIS QDs under vigorous stirring. The mixture was then heated to 100 °C for 30 min. Finally, the solution was concentrated to 10 mL by evaporation of the solvent using a rotary evaporator, purified, using isopropanol to precipitate the particle, and redispersed in 10 mL deionized water.

#### Shell synthesis CS2

For the ZnS shelling procedure, a solution of 560 µL 0.5 M aqueous GSH, 560 µL 5.0 M NH_4_OH, and 560 µL 1.0 M (containing 0.01 M HNO_3_) aqueous solution Zn(OAc)_2_ was prepared under stirring. The mixture was then added to the dispersion containing the core AIS QDs under vigorous stirring. The mixture was then heated to 100 °C for 30 min, concentrated to 10 mL by evaporation of the solvent using a rotary evaporator, purified, using isopropanol to precipitate the particle, and redispersed in 10 mL deionized water.

An overview of the described procedures is shown in Fig. 1a and b:

## Results and discussion

The nucleation and growth dynamics of colloidal QDs are controlled by the chemical nature and concentration of the precursors used including the surface ligand(s) and the reaction temperature. For QD synthesis in aqueous solution, even the reagent addition sequence can influence the temporary chemical composition of the reaction mixture. This can affect QD nucleation and growth and subsequently influence the optical properties of the resulting QDs. This encouraged us to closer examine the MW synthesis approach previously established in our group^19,42^. Thereby, two different methods of reagent addition for the preparation of the AIS core and the ZnS shell were compared and the influence of four different thiol ligands was assessed. The optical properties of the resulting QDs were then evaluated and parameters like the spectral position of the PL maximum and its spectral bandwidth (full width at half maximum (FWHM)) as well as PL QY, and PL decay kinetics were used to compare and assess the influence of the different methods and reagents.

**Figure 2:**
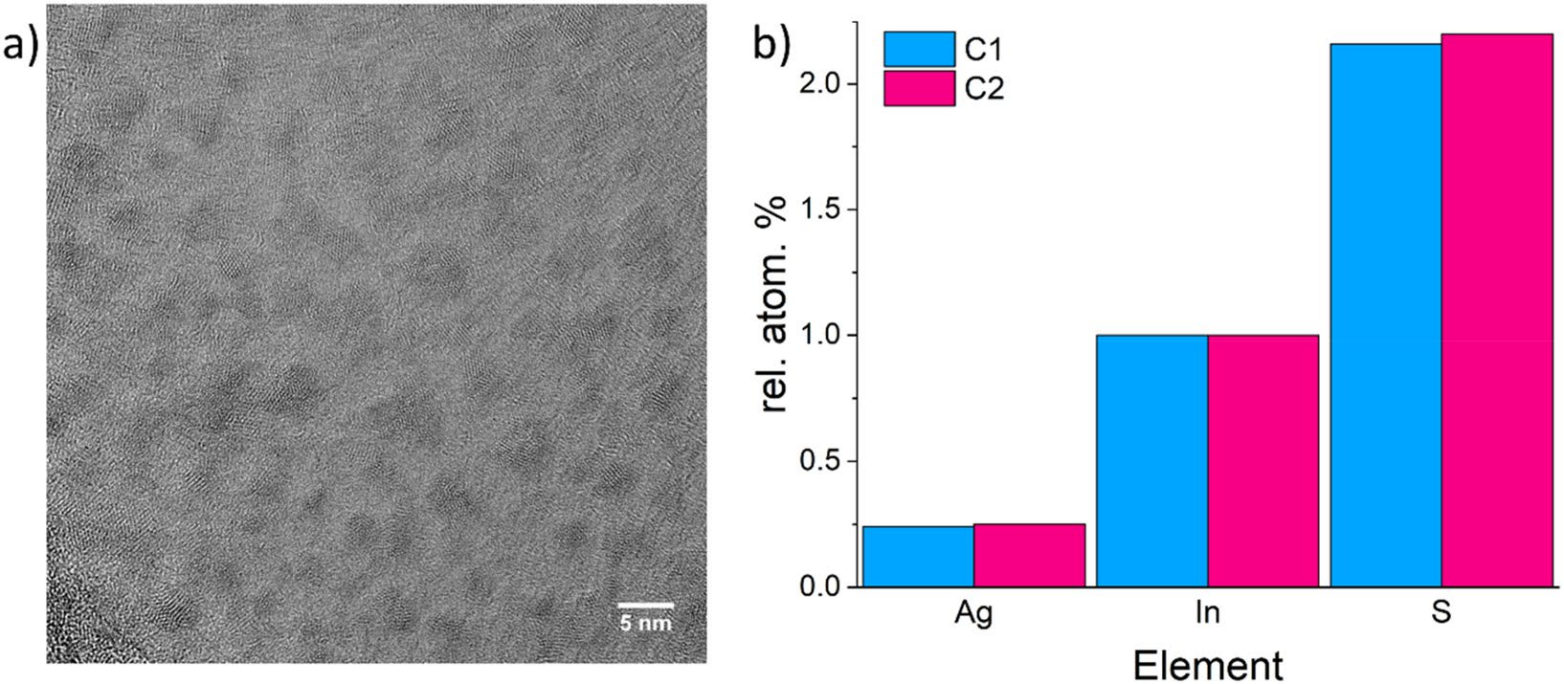
(**a**) TEM image of the AIS-GSH-capped QDs and (**b**) atomic concentration of the different constituting elements obtained by EDX analysis after normalization to the indium (In) concentration of AIS core QDs synthesized with methods *C1* and *C2*.

As starting materials for the synthesis of our AIS core QDs, we used AgNO_3_, L-reduced glutathione (GSH), NH_4_OH, citric acid (CA), InCl_3_, and Na_2_S. In previous preliminary studies, we observed that AgNO_3_ and InCl_3_ seem to be the essential reagents to start QD core formation and growth. Therefore, we investigated two different sequential orders of reagent addition: *C1* – First addition of AgNO_3_ followed by addition of GSH, NH_4_OH, CA, InCl_3_, and Na_2_S and *C2* – First addition of InCl_3_ followed by addition of GSH, NH_4_OH, CA, AgNO_3_, and Na_2_S. At first the structural properties of the prepared AIS cores were characterized. A representative TEM image of the synthesized AIS QDs is shown in Fig. 2a. This gave an average size of about 3 nm independent of the reagent addition order used. To reveal potential differences in the elemental composition of the AIS core QDs, EDX measurements were done. The results are summarized in Fig. 2b. The relative atomic percentage, normalized to the indium (In) concentration, shows a slightly higher silver (Ag) and sulphur amount for *C2* in comparison to *C1*.

**Figure 3:**
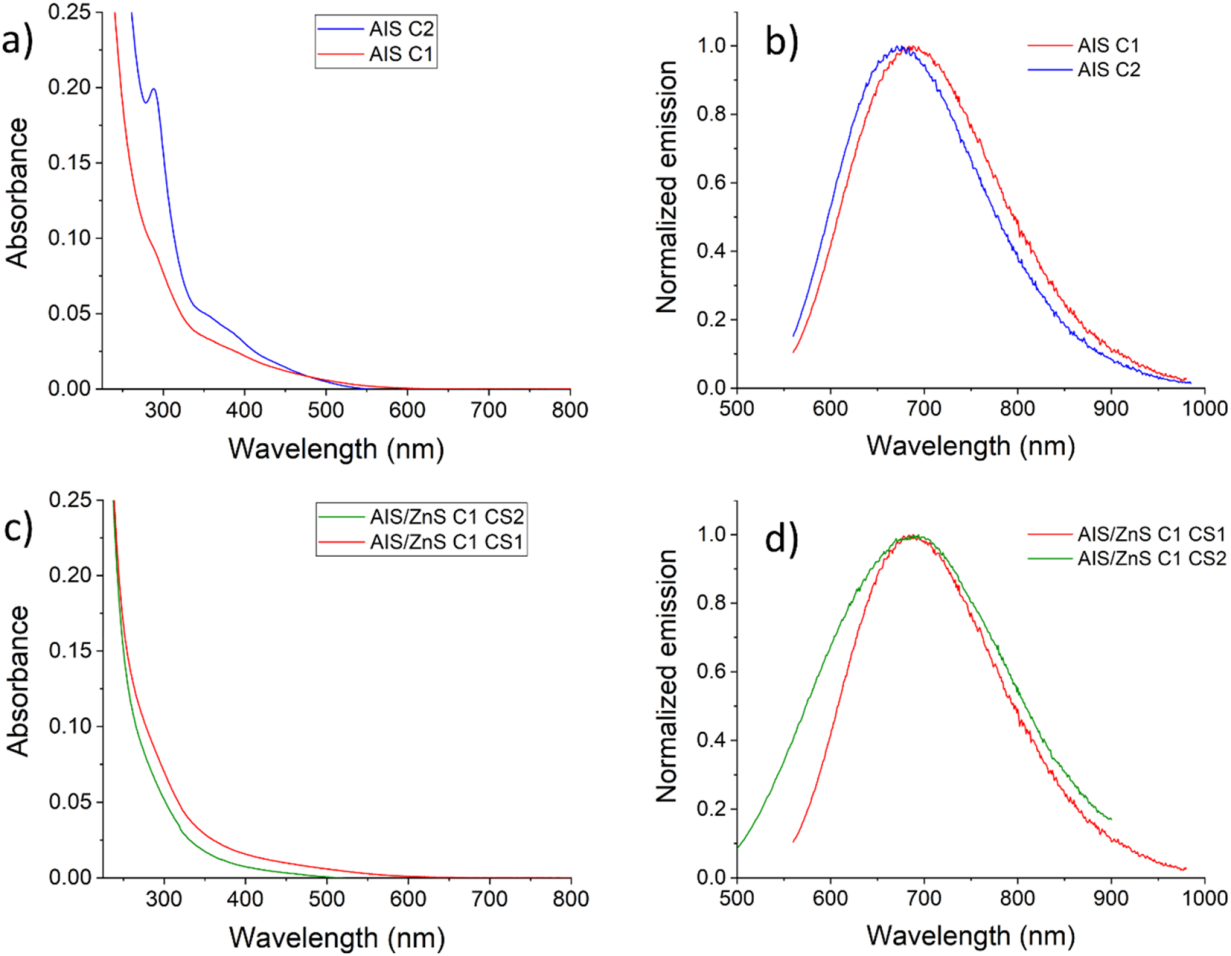
Absorption spectra and normalized emission spectra of AIS core QDs (**a**, **b**) and of Zn treated AIS/ZnS QDs (**c**, **d**) synthesized with method *C1* (red) or *C2* (blue) for core QDs and *C1-CS1* (red) and *C1-CS2* (green) for Zn treated QDs.

Next, we performed steady-state and time-resolved luminescence measurements to characterize the optical properties of the QDs obtained by methods *C1* and *C2*. The absorption spectrum of the AIS core QDs prepared by the first method shows a monotonous increase of the absorption without prominent excitonic features, which is typical for ternary QDs (see Fig. 3a)^42^. Interestingly, the absorption spectrum of the core QDs obtained with method *C2* exhibits a sharp peak at 290 nm and a broad peak at 375 nm. Since the absorption measurements of the core QD samples were done without further sample purification, the absorption feature at 290 nm may point to the formation of complexes during QD preparation. The broad feature at 375 nm could indicate the formation of a second species of very small size, maybe a complex or a cluster^46^.

The PL spectra of the AIS cores prepared by both methods are shown in Fig. 3b. A slight blue shift of the PL maxima from 690 nm (*C1*) to 670 nm (*C2*) was observed together with a narrowing of the spectral bandwidth from 200 to 170 nm for the *C2* method in comparison to the *C1* approach. Although a reduction in PL spectral bandwidth could point to a reduced number of defect states, with a value of 38%, the PL QY resulting for method *C1* is higher than that obtained by method *C2*, amounting to 28%. Time-resolved PL measurements reveal multiexponential PL decay kinetics in both cases as to be expected from ternary QDs. The PL decay kinetics were fitted with three exponents and the amplitude-weighted average decay time was calculated using equation S1. The average amplitude-weighted lifetimes of the core QDs prepared with method *C2* exhibit a shorter decay time of 427 ns in comparison to the QDs made with method *C1*, the PL of which decayed with a lifetime of 544 ns (see Supporting Information (SI), Table S1). The shorter PL decay time observed for the latter reflects the trend observed in the PL QY values. Possibly, the higher sulphur concentration of these QDs could lead to a higher number of hole traps on the QD surface, favouring a reduction in PL.

To explain the obtained results, we have to a look into the used choice of precursors. Having a closer look at the different chemicals employed in this reaction, the key point seems to be the chelation effect of the citric acid. Ag and In are present as nitrates due to the use of nitric acid in the In chloride solution, which is mandatory to prevent the instant precipitation of In hydroxide. These precursors rapidly react with citric acid at room temperature to Ag citrate and In citrate, which are both water soluble in the presence of an ammonia solution and at a basic pH value. This reaction is competitive, and the order of addition of the reagents can play a major role regarding the chemical form/nature in which the precursors are present in the subsequent reduction with Na_2_S. If Ag nitrate is added first, it can form a complex with citric acid but also with the GSH in the solution in the form of a diagonal coordination complex chain (-S(G)-Ag–S(G)-)^47^. The later added In nitrates need to compete with the Ag citrate and thus, a higher amount of free In nitrate should be present when Na_2_S is added. Therefore, when the initial Ag_2_S intermediate is formed, a faster cation exchange with the In ions can occur leading to the slight difference in QD elemental composition. This could be the reason for the higher PL QY and longer PL lifetime found for AIS QDs prepared with method *C1*. When In nitrate is added first, most likely In is nearly completely complexed by citric acid. Upon addition of Na_2_S, the release of In ions from the In citrate complex is then delayed. This can result in a slower incorporation of In ions into the Ag_2_S intermediate. This could explain the slightly higher amount of Ag found in the EDX measurements.

To increase the chemical stability and preserve the PL properties during post-modifications steps such as ligand exchange or surface functionalization with other molecules like targeting ligands, core QDs are usually coated with a second inorganic passivation shell made from a semiconductor. One of the most frequently employed shelling materials is ZnS. The surface ligands can also act as a sulphur source in the subsequent shelling step yielding AIS/ZnS QDs^48,49^. With the overall goal of assessing the influence of the sequential reagent addition, we also used two different shelling procedures for the preparation of the AIS/ZnS QDs. In the *CS1* approach, the ligand GSH, NH_4_OH, and Zn acetate were sequentially added while for the *CS2* method all reagents were premixed in a separate flask and then added to the reaction mixture. To explore solely the influence of the shelling procedure on the PL features of the resulting QDs, we chose cores prepared by method *C1* as these nanocrystals exhibited better PL properties. As shown in Fig. 3c, the absorption spectrum of the Zn-treated AIS QDs obtained by the *CS1* shelling approach closely resembles the core absorption spectrum provided in Fig. 3a. When using a premixed solution for ZnS shelling as done for shelling approach *CS2*, the absorption spectrum of the resulting QDs is blue shifted. This can point to a partial dissolution of the core QDs during the *CS2* shelling procedure^19,25^. The AIS/ZnS QDs obtained by both shelling procedures also reveal differences in their PL emission spectra summarized in Fig. 3d. Whereas the broad PL emission spectrum of the AIS/ZnS QDs obtained by method *CS1* closely matches with the PL spectrum of the core AIS QDs, the AIS/ZnS QDs prepared with method *CS2* reveal a considerable broadening of the emission band. This points to an increase in the size distribution during shelling caused by Ostwald ripening of the nanoparticles^50–52^. After the shelling procedure, for both methods, a significant increase in PL QY and the average decay times was observed. Shelling with method *CS1* results in a PL QY of 60% and PL decay time of 750 ns and method *CS2* provided QDs with a PL QY of 65% and a PL decay time of 673 ns. The major difference between both shelling methods is the precomplexation of the Zn precursor for method *CS2*. By premixing GSH and ammonia, the thiol group is reduced to a thiolate, which can then form a coordinative bond to the Zn ions added to the solution. Thereby, the sequence of reagent addition can play a role as the mixing of ammonia and Zn nitrate, formed in the presence of nitric acid in the Zn acetate solution, in the absence of GSH leads to the formation of a white precipitate, most likely Zn hydroxide, which is poorly soluble in water. The formation of Zn hydroxide is prevented for method *CS1* by the strong dilution of ammonia in the reaction mixture. When comparing methods *CS1* and *CS2,* basically, the reactivity of Zn nitrate and the Zn-GSH complex are compared regarding AIS core shelling. Here, also the instant heating of the reaction solution by MW radiation has to be considered, which prevents a heat gradient over time and makes the Zn ion release the limiting factor. Zn ion release from the Zn-GSH complexes formed in the case of method *CS2* is slower and thus the core passivation reaction is slowed down, which can subsequently enable Ostwald ripening of the AIS cores. In contrast, Zn nitrate used for shelling method *CS1* allows a fast release of Zn ions. This results in a better passivation of the AIS core surface, preventing core ripening. This is supported by the unaffected spectral width of the PL emission band of the shelled AIS QDs. Although both shelling methods lead to an increase in PL QY and high PL QY values, TEM analysis and the comparison of the diffraction pattern of the core material before and after shelling showed no significant increase in QD size and similar orthorhombic diffraction pattern. In addition, the fast Fourier transform (FTT) pattern suggest that some AIS QDs showed a tetragonal phase after Zn treatment, our so-called shelling procedure equals more a Zn passivation of the surface of the AIS cores than the formation of core/shell nanocrystals with tight and closed ZnS surface passivation shells (see SI, Fig. S1)).

Subsequently, we applied ZnS shelling procedures *CS1* and *CS2* to AIS core QDs prepared according to method *C2*. However, in this case, the shelling procedure *CS1* led to a strong agglomeration and aggregation of the AIS QDs and eventually QD precipitation. Contrary, in the case of the *CS2* shelling method, no QD agglomeration was observed. The absorption and emission spectrum of the resulting AIS/ZnS QDs closely match with those of the AIS core with no signs of additional QD ripening or dissolution (see SI, Fig. S2). With this shelling method, a PL QY of 62% was achieved. This value is identical with the PL QY value of the AIS/ZnS QDs prepared by the *C1-CS1* procedure. The average PL lifetime of the AIS/ZnS QDs amounted to 848 ns, exceeding that of the AIS/ZnS QDs made by the *C1-CS2* method. A comparison of the spectroscopic properties is given in the SI in Table S1. Apparently, the AIS cores formed by method *C2* have a different surface reactivity than those resulting from procedure *C1*. However, we can only speculate whether the formed complexes, possibly contributing to the absorption spectrum of the *C2* cores as mentioned in a previous section, could limit the colloidal stability of the *C2* cores and addition of the Zn-GSH complex is required to achieve surface passivation. Surprisingly, the Zn content of the QDs prepared with the *C2-CS2* method is higher than the QDs prepared with the *C1-CS1* method. This could also contribute to the similar PL QY values obtained after Zn treatment, overcoming the initially lower PL QY of the core AIS QDs prepared with method *C2* (see SI, Fig. S3). Nevertheless, the Zn amount is still too low to justify closed ZnS shell on the AIS QDs.

### Influence of precursor concentration

Aiming for an optimization of the NIR PL of the AIS/ZnS QDs, we subsequently varied the Ag:In ratio of the precursors for AIS core synthesis. Earlier studies showed that the elemental composition of the ternary QDs can strongly influence their optical properties and is the most effective way to tune the spectral position of the PL maximum and the PL intensity^19,25,53,54^. For example, Moodelly et al*.* reported that a deficiency of Ag results in higher PL intensities and PL QY values, which was attributed to an increased amount of Ag vacancies in the AIS crystal structure that act as acceptor states for the generation of PL^55,56^.

**Figure 4:**
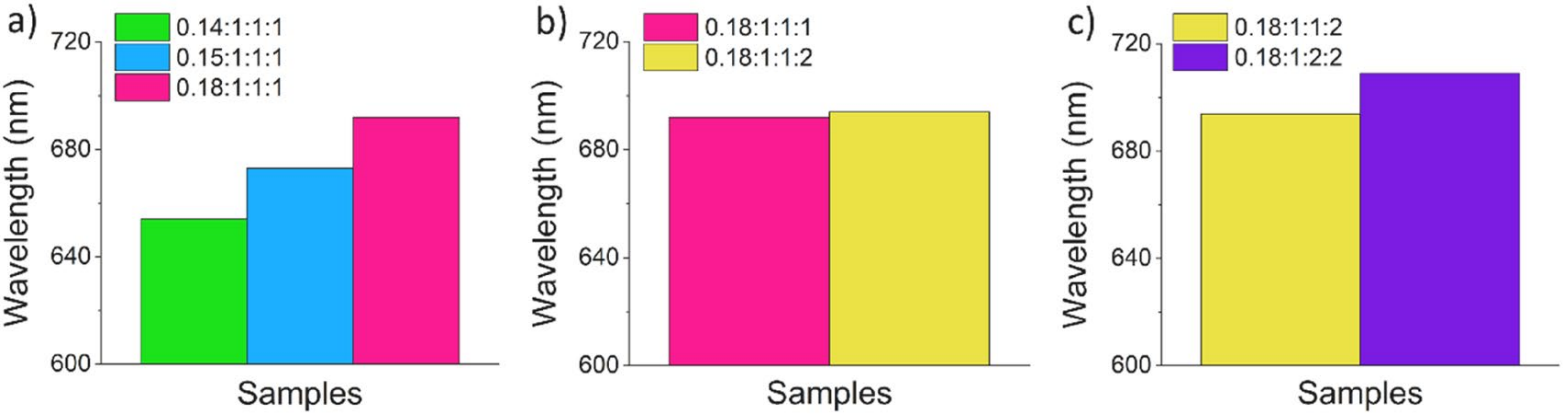
PL maximum wavelength of AIS/ZnS QDs synthesized with the capping agent GSH (0.5 M) using different ratios of Ag:In:S:Zn. (**a**) Variation of the Ag(I) amount (Ag:In:S:Zn), (**b**) variation of the Zn(II) amount (Ag:In:S:Zn), and (**c**) variation of the S amount (Ag:In:S:Zn). The PL spectra are shown in the SI, Fig. S5.

For this purpose, we synthesized AIS/ZnS QDs using methods *C1* and *CS1* for the preparation of the AIS core and the shelling procedure, respectively, and increased the Ag:In ratio from 0.14:1 to 0.18:1 (see Fig. 4a–c and SI, Table S2). Changes in the reagent concentration did not affect the absorption spectra of the samples (see SI, Fig. S4a), yet only the PL properties. With increasing Ag:In ratio, the PL maximum was shifted from 655 to 690 nm and the PL QY of the AIS core increased from 26 to 37%. The red shift can be attributed to a lower degree of Ag deficiencies, which is associated with a band gap narrowing^56^. The observed increase in PL QY with increasing Ag concentration was not yet reported in the literature. This effect can be ascribed to the MW assisted approach used here, which seems to lead to a better distribution of the donor and acceptor states within the resulting QDs than observed for AIS QDs prepared by conventional heating methods. Furthermore, the often-observed diffusion of Zn ions into the AIS cores that accounts for a blue shift of the PL emission band, did not occur in our case. This provides a clear hint that the Zn ions are located at the particle surface. Zn treatment results in a PL QY of about 60% for all AIS/ZnS QDs. Furthermore, the spectral width of the PL band slightly increased from 190 nm (550 meV) to 210 nm (548 meV) for the core/shell QDs with increasing Ag:In ratio.

Subsequently, AIS/ZnS QDs prepared with an Ag:In ratio of 0.18:1 were used to explore possibilities to further red shift the PL maximum of these ternary QDs by changes in the Zn and S concentration during the shelling procedure. As shown in the SI (Fig. S4b,c), the absorption spectra of the samples and their PL properties changed only slightly. Except for a very small red shift of the PL band of the AIS QDs obtained with a doubling of the initially employed Zn concentration, we observed no further changes in the PL features (see Fig. 4b). Also the increased Zn concentration did not significantly improve the PL QY (see SI, Table S2). Doubling the S concentration caused slight changes in the absorption properties of the QDs and a red shift of the PL maximum from 690 to 710 nm and a slight increase of the FWHM from 205 nm (535 meV) to 220 nm (542 meV) (see Fig. 4c). The PL QY of about 60%, however, remained constant (see SI, Table S2).

### Influence of thiol ligands on the synthesis of AIS core and AIS/ZnS QDs

Ligands play an important role during QD synthesis, affecting QD seed formation and growth, and subsequently also for the surface chemistry of the resulting QDs^26,57^. Ligands can influence nanocrystal nucleation by varying precursor reactivity and growth kinetics, thereby facilitating or hampering the adsorption of monomers on the surface of the growing QDs. This can influence nanocrystal size, size distribution, and shape as well as functional properties such as PL. In addition, thiol ligands can act as sulphur source during AIS QD formation at elevated temperatures.

**Figure 5:**
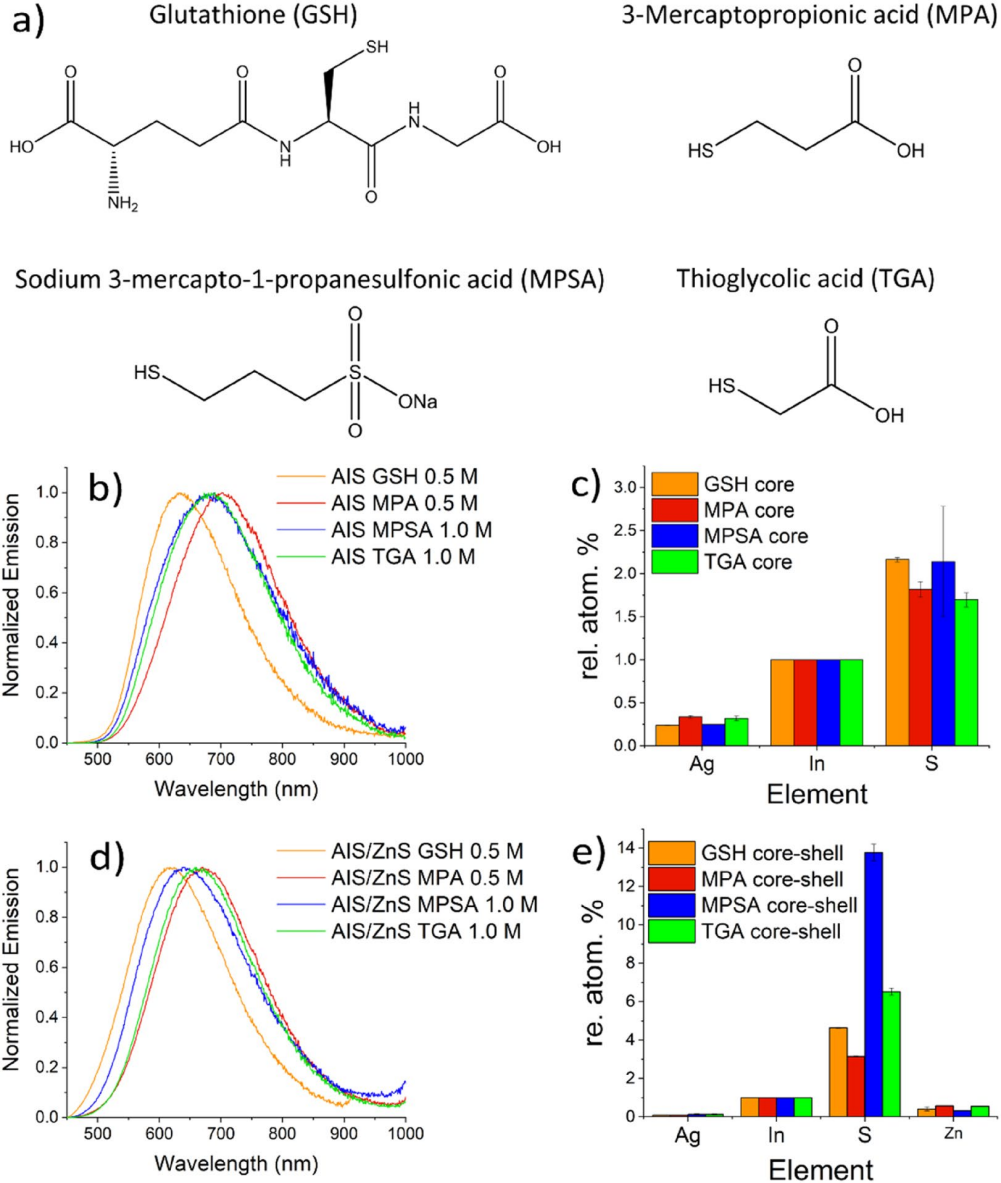
(**a**) Chemical structures of thiol ligands L-reduced glutathione (GSH), 3-mercaptopropionic acid (MPA), sodium 3-mercapto-1-propane sulfonic acid (MPSA), and thioglycolic acid (TGA). Normalized emission spectra and relative element concentrations of the AIS core QDs (**b**, **c**) and AIS/ZnS QDs (**d**, **e**).

To assess the influence of the surface stabilizing thiol ligands on the PL features of AIS QDs, we synthesized AIS/ZnS QDs with four widely used thiol ligands varied in concentration, always using a precursor ratio of Ag:In:S:Zn 0.18:1:1:2. Thiol ligands explored included chiral L-reduced glutathione (GSH), 3-mercaptopropionic acid (MPA), sodium 3-mercaptopro-1-propanesulfonic acid (MPSA), and thioglycolic (TGA). The chemical structures of the thiol ligands are shown in Fig. 5a. Criteria for ligand choice included ligand size and ligand structure, i.e., the presence of additional functionalities like carboxylic groups. These groups can be deprotonated at pH values exceeding 5.5 (pK_a_ of carboxylic acid groups of about 5.5), thereby also contributing to the electrostatic stabilization of the QDs at neutral and basic pH values. Moreover, the presence of these functionalities can be exploited for the covalent coupling of targeting bioligands to the QD surface. Compared to the relatively small monodentate thiols MPA and TGA bearing a single carboxylic group, GSH shows a more complex structure with a thiol group and two carboxylic groups. MPSA was chosen as an interesting alternative to thiol ligands bearing carboxylic groups as its sulfonic acid group also provides water dispersibility. Such sulfonic acid groups are often employed to render organic fluorophores like cyanine dyes water soluble without introducing additional functionalities that can be deprotonated in the commonly used pH regime^58–61^.

For this ligand comparison, the AIS cores were always prepared with method *C1* and the Zn treatment was made by procedure *CS1*. The resulting core QDs were then structurally and spectroscopically characterized before and after Zn treatment. Interestingly, for a ligand concentration of 0.5 M, the AIS core QDs prepared with TGA and MPSA were colloidally stable but after Zn treatment with the same ligand, the resulting AIS/ZnS QDs started to precipitate. However, by increasing the ligand concentration to 1 M, colloidally stable TGA- or MPSA-capped AIS/ZnS QDs could be obtained. For the short ligand TGA, this suggests partial decomposition during the shelling procedure, thus requiring a higher amount of TGA to stabilize the resulting QDs. This could also explain the increased sulphur amount after shelling which follows from Fig. 5d. As MPSA has a similar length than MPA, that yielded stable QDs, this effect seems to originate from the sulfonic acid group solely present in this ligand. One possible explanation could be here the decomposition of the MPSA ligand during the shelling procedure with our MW assisted approach. This assumption is supported by the considerable increase in the amount of sulphur in the QDs after the shelling step as shown in Fig. 5d.

For the ligands MPA and TGA, the absorption spectra of the core QDs shown in the SI (Fig. S6a) reveal the typical featureless shape of ternary QDs, while in the case of GSH and MPSA, an additional band at 290 nm is observed (SI, Fig. S6b). This band could originate from a complex species formed as intermediate during the initial reaction period as mentioned before. Also, the otherwise unstructured absorption spectra of AIS core QDs capped with MPA, TGA, and MPSA show a small absorption band at 353 nm, which disappeared during the prolonged heating step employed for the shell synthesis. One possible explanation of this feature could be the intermediate formation of small clusters during AIS core synthesis. The normalized emission spectra of the AIS and AIS/ZnS QDs synthesized in the presence of the different ligands are summarized in Fig. 5b and d. The PL maximum of the GSH-capped AIS core QDs is located at 632 nm, while the PL maxima of the AIS QDs obtained with the other smaller ligands are red shifted by about 60 nm. The slightly higher Ag content of the AIS QDs when using the short chain thiol ligands TGA and MPA (see EDX data, Fig. 5c) could suggest a slightly reduced exchange with In ions resulting in the observed red shift in PL. This can, however, not explain the PL shift for AIS QDS prepared with MPSA. The comparable elemental distribution derived from the EDX measurements of the AIS QDs synthesized with GSH and MPSA can point to a specific influence of the sulfonic acid group of MPSA on the PL properties. Here, more systematic studies with a broader range of thiol ligands containing different functional groups are required. After shell growth, the PL emission maxima for all four thiol ligands are blue shifted, yet to a different extent. This blue shift amounts to 15 nm (0.04 eV) for GSH, to 30 nm (0.08–0.09 eV) for the smaller thiol ligands TGA and MPA, and to about 60 nm (0.15 eV) (Fig. 5d) for MPSA. This shelling-induced blue shift is commonly explained by the incorporation of Zn ions into the QD core during the shelling process^62^. The difference in the magnitude of the hypsochromic shift induced upon Zn treatment does not present an indicator for the relative amount of Zn present in the core as the Zn concentration is identical for all QDs (Fig. 5e). Although the very small Zn concentration obtained after Zn treatment makes the formation of a closed ZnS monolayer shell not very plausible as previously stated, the PL QY of the core/shell systems prepared from GSH- and MPA-stabilized AIS QDs nevertheless reached high PL QY values of 60%. The TGA and MPSA-capped AIS/ZnS QDs showed smaller, yet still moderate PL QY values of 40% and 45%. The lower values obtained for the latter two ligands are ascribed to the increased sulphur concentration after Zn treatment (see Fig. 5e), apparently originating from sulphur ions released from TGA and MPSA. This could result in a sulphur rich surface favouring hole trapping and thereby PL quenching^63^. To better understand the photophysical processes involved in the PL of our AIS QDs, we correlated the amplitude weighted average PL decay times derived from the PL decay curves with the PL QY values. Although a high PL QY comes usually along with a long PL lifetime, a comparison of the PL properties of the AIS QDs made with GSH and MPA revealed that the average PL decay time of 460 ns of the AIS QDs prepared with GSH is considerably shorter than the PL decay time of 610 ns of the AIS QDs made with MPA, despite comparable PL QY values of 60%. This observation can point to differences in the radiative recombination pathways in these materials. Hence, to obtain a closer insight into the photophysics of these AIS QDs, we fitted the PL decay curves to derive individual PL decay times, being aware that the discussion of individual decay times is still debated for QDs. Nevertheless, this can provide a first hint for possible explanations of the differences in the radiative recombination pathways. When comparing the individual decay times, the fractions of the decay time components revealed considerable differences for the GSH- and MPA capped AIS QDs after Zn treatment (see SI, Table S3). For the GSH capped AIS QDs, the contribution (fraction) of the short PL component, which is related to PL involving surface states, is smaller than that of the second longest decay time, which is attributed to PL from core states^64^. This is the opposite for the MPA-capped AIS QDs. This points to an enhanced PL surface quenching of the GSH-capped QDs, most likely caused by the higher sulphur concentration of these QDs, as sulphur species can act as hole traps (see Fig. 5e). Due to the smaller PL shift after Zn treatment, which suggests less Zn in the AIS QD cores, the core states are less affected in the case of the GSH-capped AIS QDs than for the MPA capped QDs. This seems to partly compensate for the increased sulphur-induced PL quenching. A similar trend is observed for TGA- and MPSA-capped QDs, that show short average PL decay times of 540 ns and 400 ns, respectively. As these two AIS QDs revealed a stronger PL blue shift after Zn treatment, the core states are apparently more affected than those in GSH-capped AIS QDs. This could explain the lower PL QY values.

## Conclusion and outlook

Aiming for the identification of reaction conditions providing high quality heavy metal-free semiconductor quantum dots (QDs) with visible and near infrared (NIR) emission, we systematically assessed several parameters of the microwave-assisted (MW) synthesis of AIS and AIS/ZnS semiconductor quantum dots (QDs) in water using relatively green synthesis conditions. As tools for the evaluation of the resulting QDs, their chemical composition and particularly their photoluminescence (PL) properties were utilized. Our results show that the sequential order, the precursor concentration, and the choice of the thiol capping ligand all have a significant influence on the optoelectronic properties of the synthesized QDs. Under optimum conditions, QDs with high photoluminescence quantum yields (PL QY) values of 60–65% were obtained. These QDs are sufficiently bright for applications as reporter in bioimaging studies and the design of sensors. Their application in luminescent solar concentrators, however, ideally requires higher PL QY close to one^2^. Whereas the precursor concentration and the ligand choice can be expected to have a major influence on the quality of the QDs, the significance of the sequential order of reagent addition was surprising and is underexplored for this type of QD.

Our results obtained with the four thiol ligands glutathione (GSH), 3-mercaptopropionic acid (MPA), sodium 3-mercaptopro-1-propanesulfonic acid (MPSA), and thioglycolic (TGA) also underline the significant effect of the ligand on the optoelectronic properties of AIS QDs, particularly on their PL features. Based on the PL properties of our AIS core and AIS/ZnS QDs, MPA and GSH are the best choice for realizing NIR emitting QDs with a high PL QY. Due to the higher ligand density on MPA-capped QDs compared to their GSH-stabilized counterparts, these QDs can be advantageous for all applications where the QD concentration considerably changes, i.e., due to inherent dilution. This can be the case, e.g., for cell studies. The use of the chiral ligand GSH can be beneficial for the preparation of chiral QDs enabling the correlation of the absorption and PL features of QDs with circular dichroism (CD) studies^20^. In the future, more detailed studies of the formation of AIS/ZnS QDs are necessary to identify the chemical nature of the monomers, complexes, and possibly even clusters formed during the course of this reaction.

## Supplementary Information

Supplementary Information.

## Data Availability

The datasets used and/or analysed during the current study are available from the corresponding author on reasonable request.
