# Supplementary material for: Assessing the influence of microwave-assisted synthesis parameters and stabilizing ligands on the optical properties of AIS/ZnS quantum dots
Source: Sci Rep. 2022 Dec 20;12:22000. doi: 10.1038/s41598-022-25498-3 (PMC9767924; doi:10.1038/s41598-022-25498-3)
Supplement: Supplementary file 1 — Supplementary Information. [file 41598_2022_25498_MOESM1_ESM.pdf]

Supporting information

Assessing the influence of microwave-assisted synthesis parameters and stabilizing ligands on the optical properties of AIS/ZnS quantum dots

Lorena Dhamo,[a,b,#] K. David Wegner,[a,#] Christian Würth,[a] Ines Häusler,[b] Vasile-Dan Hodoroaba[c] and Ute Resch-Genger*[a]

a) Division Biophotonics, Federal Institute for Materials Research and Testing (BAM), 12489 Berlin, Germany
b) Departments of Physics, Humboldt Universität zu Berlin, 12489 Berlin, Germany
c) Departments of Physics, Humboldt Universität zu Berlin, 12489 Berlin, Germany
d) Department of Materials Protection and Surface Technologies, Federal Institute for Materials Research and Testing (BAM), 12203, Berlin, Germany
#: These authors contributed equally.

Calculation of amplitude-weighted ($<\tau>_{amp}$) average lifetimes:

$$< \tau >_{amp} = \frac{\sum_n A_n \tau_n}{\sum_n A_n} \qquad \text{S1}$$

where A_n are the amplitudes and τ_n the lifetimes of the respective decaying species.

Table S1: Quantum yield (QY), average amplitude-weighted decay time, and fullwidth at half maxima (FWHM) of AIS and AIS/ZnS QDs.

Sample	QY (AIS-AIS/ZNS)	τ_{amp} [ns] (AIS-AIS/ZnS)	FHWM (AIS-AIS/ZnS)
**C1 CS1**	38 – 60%	544 – 750 ns	200 – 220 nm
**C1 CS2**	38 – 65%	597 – 673 ns	216 – 229 nm
**C2 CS2**	28 – 62%	427 – 848 ns	169 – 165 nm

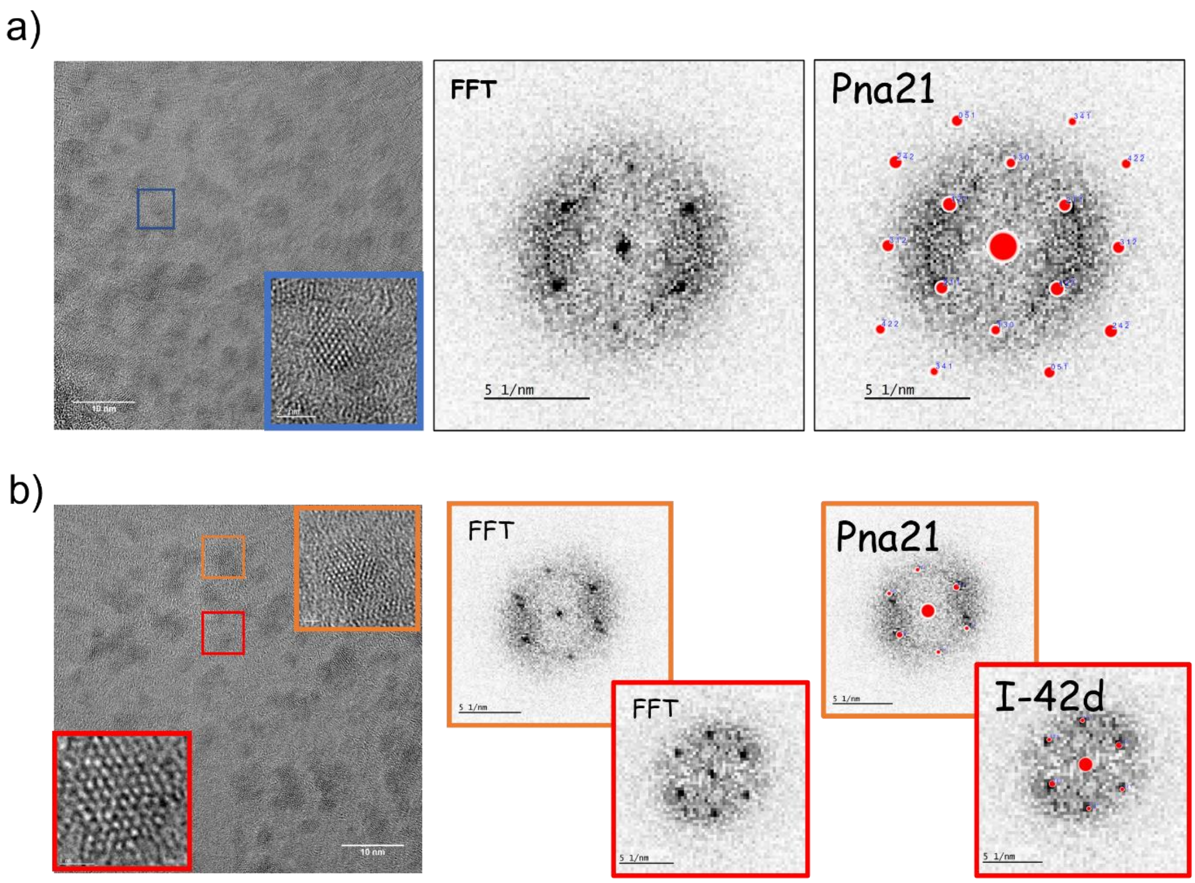

Figure S1: HRTEM images of core (**a**)) and Zn treated AIS/ZnS QDs (**b**)) capped with GSH. The fast Fourier transform (FTT) patterns suggest an orthorhombic diffraction pattern (Pna21) for core AIS QDs and a mixture of tetragonal (I-42d) and orthorhombic diffraction patterns for Zn treated AIS/ZnS QDs.

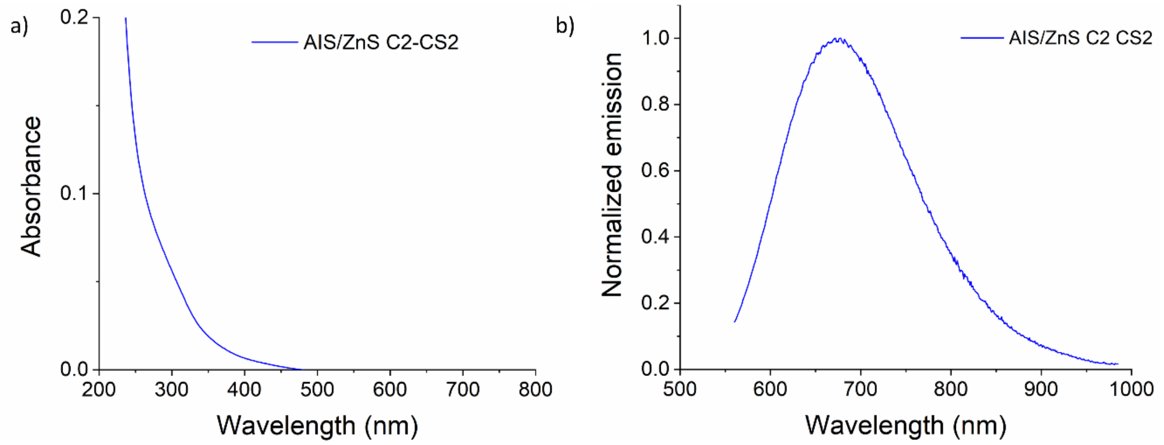

Figure S2: Absorption (**a**)) and emission spectrum (**b**)) of AIS/ZnS QDs synthesized with the method C2 for core QD and CS2 for Zn treatment.

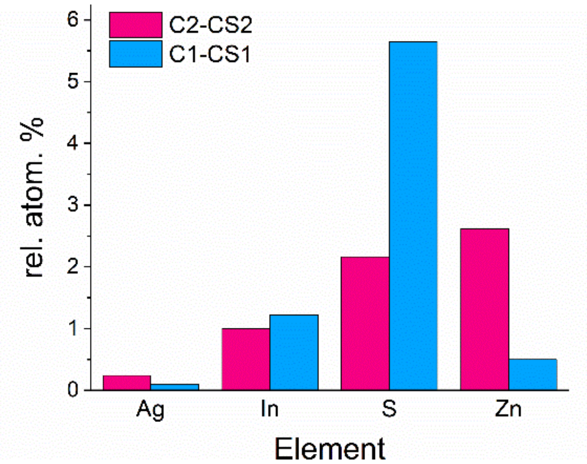

Figure S3: Atomic concentrations of QDs prepared using method C2 for the core and CS2 for the Zn treatment (red) and using method C1 for the core and CS1 for the Zn treatment (blue).

Table S2: Quantum yield (QY) and the full width at half maxima (FWHM) of the emission band for AIS/ZnS QDs synthesized using different Ag:In:S:Zn element ratios.

Component's Ratio					QY (AIS-AIS/ZnS)	FWHM (AIS-AIS/ZnS)
$AgNO_3$	$InCl_3$	Na_2S	$Zn(OAc)_2$	GSH		
**0.14**	1	1	1	7	26 – 56%	155 – 190 nm
**0.15**	1	1	1	7	30 – 55%	155 – 197 nm
**0.18**	1	1	1	7	37 – 58%	180 – 210 nm
**0.18**	1	1	2	7	36 – 61%	175 – 205 nm
**0.18**	1	2	2	7	37 – 57%	220 – 220 nm

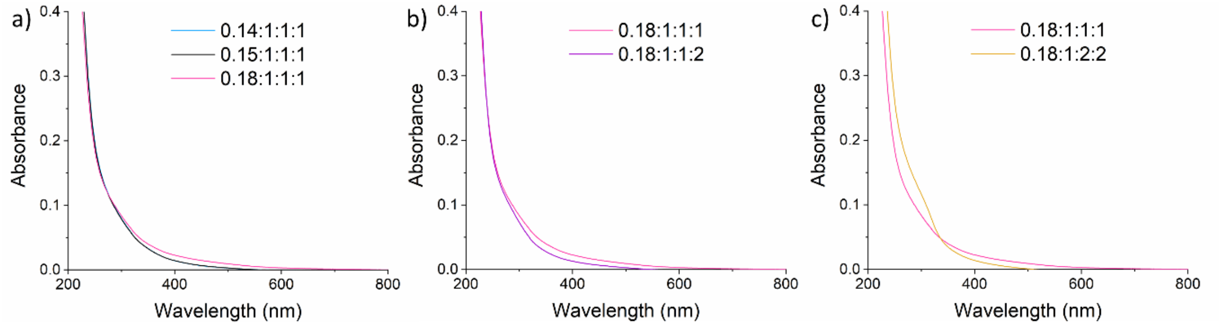

Figure S4: Absorption spectra of AIS/ZnS QDs 0.5 M GSH-capped synthesised with different ratio of the components: Ag:In:S:Zn with Ag amount varied (**a)**), Ag:In:S:Zn with Zn varied (**b)**), and Ag:In:S:Zn with S amount varied (**c)**).

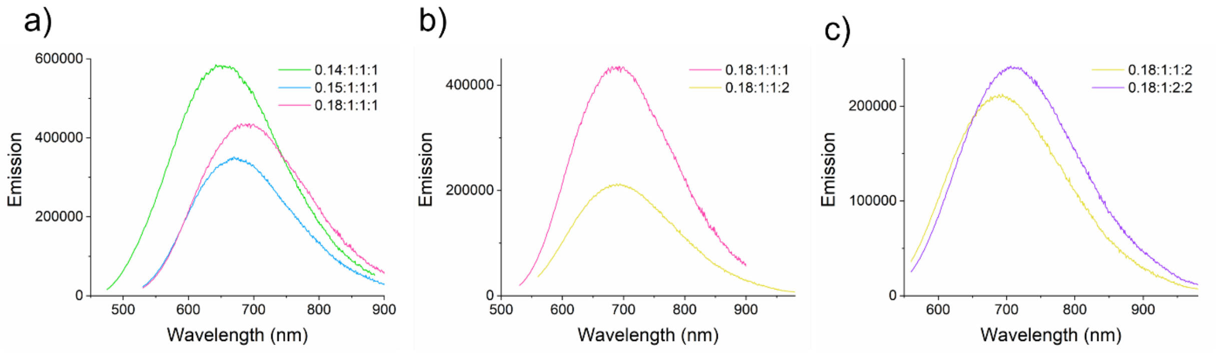

Figure S5: PL spectra of AIS/ZnS QDs synthesized with the capping agent GSH (0.5 M) using different ratios of Ag:In:S:Zn. **a)** Variation of the Ag(I) amount (Ag:In:S:Zn), **b)** variation of the Zn(II) amount **(**Ag:In:S:Zn), and **c)** variation of the S amount (Ag:In:S:Zn).

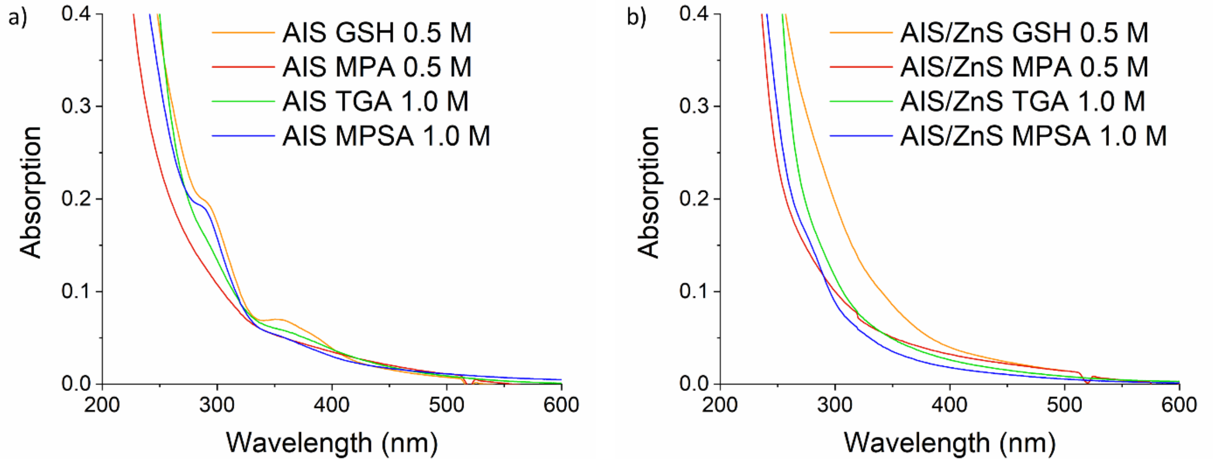

Figure S6: Absorption spectra of AIS (**a)**) and AIS/ZnS (**b)**) QDs using the ligands GSH-(yellow), MPA-(red), MPSA-(blue), and TGA-capped (green) during the synthesis.

Table S3: Measured PL decay times (resulting from tri-exponential fits of the PL decay curves) as well as amplitude-weighted and intensity-weighted average decay times of the AIS and AIS/ZnS QDs with GSH, MPA, MPSA and TGA as capping ligands.

**QD sample**	**τ (ns) (with fractions in brackets)**	**τ_{amp}[ns]**	**τ_{int} [ns]**
**GSH AIS**	77 (31%)<br>297 (45%)<br>648 (24%)	310 ± 5	450 ± 5
**GSH AIS/ZnS**	204 (31%)<br>490 (62%)<br>1232 (7%)	460 ± 5	605 ± 5
**MPA AIS**	238 (52%)<br>702 (45%)<br>2397 (3%)	505 ± 5	810 ± 5
**MPA AIS/ZnS**	332 (59%)<br>857 (39%)<br>3512 (2%)	610 ± 5	1050 ± 5
**TGA AIS**	119 (39%)<br>454 (53%)<br>1149 (8%)	385 ± 5	600 ± 5
**TGA AIS/ZnS**	306 (65%)<br>837(33%)<br>3578 (2%)	540 ± 5	1000 ± 5
**MPSA AIS**	93 (43%)<br>363 (55%)<br>755 (2%)	340 ± 5	500 ± 5
**MPSA AIS/ZnS**	175 (40%)<br>475 (52%)<br>1039 (8%)	400 ± 5	540 ± 5
